# Organization of ATP-gated P2X1 receptor intracellular termini in apo and desensitized states

**DOI:** 10.1085/jgp.201812108

**Published:** 2019-02-04

**Authors:** Alistair G. Fryatt, Sudad Dayl, Anastasios Stavrou, Ralf Schmid, Richard J. Evans

**Affiliations:** 1 Department of Molecular and Cell Biology, University of Leicester, Leicester, UK; 2 Department of Chemistry, College of Science, University of Baghdad, Baghdad, Iraq; 3 Leicester Institute of Structural and Chemical Biology, University of Leicester, Leicester, UK

## Abstract

The human P2X1 receptor (hP2X1R) is a trimeric ligand-gated ion channel opened by extracellular ATP. The intracellular amino and carboxyl termini play significant roles in determining the time-course and regulation of channel gating—for example, the C terminus regulates recovery from the desensitized state following agonist washout. This suggests that the intracellular regions of the channel have distinct structural features. Studies on the hP2X3R have shown that the intracellular regions associate to form a cytoplasmic cap in the open state of the channel. However, intracellular features could not be resolved in the agonist-free apo and ATP-bound desensitized structures. Here we investigate the organization of the intracellular regions of hP2X1R in the apo and ATP-bound desensitized states following expression in HEK293 cells. We couple cysteine scanning mutagenesis of residues R25-G30 and H355-R360 with the use of bi-functional cysteine reactive cross-linking compounds of different lengths (MTS-2-MTS, BMB, and BM(PEG)_2_), which we use as molecular calipers. If two cysteine residues come into close proximity, we predict they will be cross-linked and result in ∼66% of the receptor subunits running on a Western blot as dimers. In the control construct (C349A) that removed the free cysteine C349, and some cysteine-containing mutants, cross-linker treatment does not result in dimerization. However, we detect efficient dimerization for R25C, G30C, P358C, K359C, and R360C. This selective pattern indicates that there is structural organization to these regions in the apo and desensitized states in a native membrane environment. The existence of such precap (apo) and postcap (desensitized) organization of the intracellular domains would facilitate efficient gating of the channel.

## Abstract (precis)

The intracellular amino and carboxyl termini of the P2X1 receptor associate to form a cytoplasmic cap on the open state. Fryatt et al. use cysteine-reactive cross linking and molecular modeling to demonstrate structural organization of the intracellular termini in the apo and desensitized states.

## Introduction

Extracellular ATP acting at cell surface P2X receptors (P2XRs) plays an important role in a variety of physiological and pathophysiological conditions (Kaczmarek-Hájek et al., 2012). There are seven mammalian genes encoding P2XR subunits (P2X1-7; Surprenant and North, 2009). The expression pattern of these is tissue-specific, and it is clear that defined P2XRs have distinct roles, raising their therapeutic potential. For example, P2X1Rs are involved in thrombosis (Hechler et al., 2003) as well as neutrophil activation (Lecut et al., 2012), and blocking P2X3R activity is an effective treatment for cough (Abdulqawi et al., 2015). The P2XR subunits assemble to form homo- and heterotrimeric channels often with distinct properties in terms of agonist sensitivity and/or the time-course of the response. For example, P2X1 and P2X3Rs have EC_50_ values of ∼1 µM ATP, and evoked currents decay rapidly (<1 s) in the continued presence of agonist (Kaczmarek-Hájek et al., 2012). In contrast, P2X7Rs have millimolar sensitivity to ATP, responses that increase in amplitude to subsequent applications, and currents that do not decay during continued agonist application (Roger et al., 2010). Variations in the extracellular ligand binding region of the receptor contribute to differences in agonist sensitivity (Young et al., 2007). The first information on the molecular mechanisms regulating the time-course of responses came from work looking at splice variants of the P2X2R showing that the absence of a section of the intracellular carboxyl terminus sped the decay of ATP-evoked currents (Brändle et al., 1997; Simon et al., 1997). Studies have shown subsequently that both the amino and carboxyl termini contribute to the time-course of responses (Boué-Grabot et al., 2000), not only the rate of desensitization but also recovery from the desensitized state (Allsopp and Evans, 2011; Allsopp et al., 2013). In addition, the transmembrane domains can regulate the time-course of the response, and there is evidence that this is linked to changes in the intracellular regions (Werner et al., 1996; Allsopp and Evans, 2011).

The crystallization of the zebrafish P2X4R was a major advance and allowed a molecular understanding of a range of properties of the receptors, e.g., agonist binding and the location of the channel gate (Kawate et al., 2009; Hattori and Gouaux, 2012). However, structural information regarding the intracellular domains was still elusive due to the truncation of the intracellular regions required for crystallization. Recently, a series of structures of the hP2X3R have been published using a construct with longer intracellular regions (Mansoor et al., 2016). The hP2X3R shows rapid desensitization, but introduction of mutations in the amino terminus resulted in a receptor that showed an initial peak response to ATP that then declined to a sustained level ∼10% of the peak response (Hausmann et al., 2014; Mansoor et al., 2016). By comparing different conditions and constructs, a gating cycle of the hP2X3R has been proposed. The ATP-bound structure with the mutations in the amino terminus shows an open transmembrane channel gate and an intracellular cytoplasmic cap formed from the interaction of the amino and carboxyl termini. No structural information on the intracellular termini could be resolved in the apo or desensitized states, leading to the suggestion that these regions are flexible and disordered. However, given the complex interdigitated assembly of the amino and carboxyl termini, the question arises how the cap forms quickly and efficiently in order to give rapid channel openings. One possibility is that there is some structural organization of the intracellular regions in the apo and desensitized states but that this could not be resolved under the conditions used for crystallization of the hP2X3R.

P2XRs have been shown to interact with a range of signaling molecules and proteins that can modify channel function (e.g., Kim et al., 2001; Chaumont et al., 2008; Lalo et al., 2011, 2012). In addition, the lipid composition of the membrane also regulates channel properties (Fujiwara and Kubo, 2006; Bernier et al., 2013; Murrell-Lagnado, 2017). For the hP2X1R, the intracellular regions have been shown to be involved in this regulation (Allsopp et al., 2010; Lalo et al., 2011, 2012), suggesting that cellular regulatory factors/accessory proteins play important roles in the structural integrity of the hP2X1R. Therefore, we wanted to obtain molecular information on the intracellular regions of hP2X1Rs expressed in as native an environment as possible. To do this, we looked at the ability of membrane-permeant bi-functional cysteine reactive compounds to cross-link cysteine point mutations introduced into the intracellular juxta-transmembrane domains of hP2X1Rs expressed in HEK293 cells. These studies highlighted a pattern of cross-linking that suggests some structural organization in both the apo and desensitized states.

## Materials and methods

### Site-directed mutagenesis

Cysteine point mutations were introduced via the QuikChange mutagenesis kit (Stratagene) using the hP2X1R plasmid as the template, as described previously (Roberts and Evans, 2007). DNA sequencing (Automated ABI Sequencing Service, University of Leicester, Leicester, UK) verified the absence of coding errors and incorporation of the correct mutations in the P2X1 mutant constructs.

### Cell culture and plasmid transfection

To express the hP2X1Rs, the constructs were transfected into HEK293F cells (Invitrogen, Thermo Fisher Scientific UK Ltd; Watson et al., 2016). To transfect 100 ml of cells, 100 µg of each construct was diluted in 10 ml PBS (Sigma-Aldrich) and vortexed, and 0.2 mg 25 kD branched polyethylenimine (Sigma-Aldrich) added. This mixture was vortexed and incubated for 20 min at room temperature. This was then added to cells at a final density of 10^6^ cells per milliliter. Cells were collected after 48 h and washed in PBS (adjusted to pH 7.0 to aid cross-linker specificity) by centrifugation. The cells were resuspended in PBS and divided equally between the experimental conditions. In some experiments, HEK293 cells were transfected in six-well plates using lipofectamine (Allsopp and Evans, 2015). Cells were preincubated in either 3.2 U/ml^−1^ apyrase or 300 µM ATP (Sigma-Aldrich) for 10 min at room temperature with gentle rocking. Cross-linking experiments were performed on intact cells expressing recombinant hP2X1Rs. The cells were incubated in 300 µM cysteine-reactive cross-linker, 1,2-ethanediyl bismethanethiosulfonate (MTS-2-MTS; Santa Cruz Biotechnology), 1,4-bismaleimidobutant (BMB), or 1,8-bismaleimido-diethyleneglycol (BM[PEG]_2_; Thermo Fisher Scientific), or DMSO (vehicle control) for 30 min in the presence of apyrase or ATP at room temperature with gentle rocking. For some studies, surface hP2X1Rs were isolated using sulfo-NHS-LC-biotin (as described previously; Ennion and Evans, 2001). Any unbound cross-linker reactivity was then quenched by adding an excess volume of 10 mM cysteine in PBS. Cells were centrifuged and the pellet was lysed in buffer containing 50 mM Tris base, 150 mM NaCl, and 10 mM *N*-ethylmaleimide (NEM), pH 8.0, with 10 µl/ml^−1^ protease inhibitor cocktail (Sigma-Aldrich). The lysate was centrifuged and supernatant retained for SDS-PAGE analysis.

### Sample preparation and SDS-PAGE

Cleared supernatants were combined with an equal volume of SDS sample buffer, boiled at 95°C for 5 min, then centrifuged for 5 min. Samples and 10 µl of biotinylated protein ladder (New England Biolabs) were loaded onto 10% SDS-PAGE gels. The gels were electrophoresed at 120 V for 140 min to ensure suitable separation of any higher-molecular-weight protein complexes. The separated proteins were transferred to nitrocellulose membrane (GE Healthcare, VWR International Ltd) at 100 V for 60 min. To confirm protein transfer, membranes were stained with Ponceau S solution (Sigma-Aldrich), then washed with Tris-buffered saline with Tween 20 (TBST). Membranes were incubated overnight in 5% nonfat milk TBST at 4°C with constant agitation, then washed with TBST. Proteins were identified by using rabbit anti–P2X1 (1:500; Alomone) then horseradish peroxidase-conjugated (HRP) goat anti-rabbit (1:2,000; Sigma-Aldrich) with antibiotin HRP (1:1,000, New England Biolabs). All antibodies were diluted in 5% nonfat milk TBST, incubated for 60 min at room temperature, and washed thoroughly with TBST after each incubation. The protein/antibody complexes were visualized by chemiluminescent detection. Membranes were incubated in Amersham ECL prime (GE Healthcare) for 5 min before the excess solution was removed. The membranes were wrapped in cling film and scanned using the chemiluminescent detection settings of the Typhoon Trio+ imager (GE Healthcare).

### Patch clamp recording of hP2X1R currents

Whole cell patch voltage clamp recordings were made from HEK293 cells cotransfected with hP2X1R mutant DNA 0.45 µg and 0.05 µg of DNA for GFP. After 24 h of incubation, cells were plated onto 13 mm coverslips and left to grow in Dulbecco's Modified Eagle's Medium (DMEM) until recordings were made. Following transfer of the coverslip to the recording chamber, they were perfused continuously with an extracellular solution containing (in mM) 150 NaCl, 2.5 KCl, 2.5 CaCl_2_, 1 MgCl_2_, 10 HEPES, and 10 glucose (pH to 7.3 with NaOH). ATP (100 µM) was applied with a U-tube perfusion system. Membrane currents from fluorescent cells expressing GFP and hP2X1Rs were recorded at a holding potential of −70 mV (corrected for tip potential) using an Axopatch 200B amplifier (Molecular Devices). Data were low pass–filtered at 1 kHz, digitized at a sampling interval of 200 µs, and acquired using a Digidata 1200 analogue-to-digital converter with pClamp 9.2 acquisition software (Molecular Devices). Patch pipettes were filled with internal solution composed of 140 mM potassium gluconate, 10 mM EGTA, 10 mM HEPES, 5 mM NaCl (pH 7.3, adjusted with KOH), and had resistances in the range of 2–6 megohms.

### Densitometry analysis

ImageJ (National Institutes of Health) was used to measure any changes in molecular weight following cross-linker exposure. Briefly, the whole length of each lane of the membrane image was analyzed, and pixel density for each band present was measured. The percentage of the total pixel density of the band at ∼100–120 kD was then calculated as the percentage of the P2X1R subunits that had dimerized. Data collected from densitometry analysis are shown as mean ± SEM with histograms plotted using GraphPad Prism 7 (GraphPad Software). Any differences between the means were determined by one-way ANOVA with the Bonferroni posttest *(n* ≥ 3 for all average data). Actual P values from the figures are given in Table S1.

### Molecular modeling

Models of the hP2X1R in the closed state including the intracellular region were built using a hybrid modeling approach in ROSETTA v3.4 (Das and Baker, 2008). The extracellular and transmembrane regions of the hP2X1R were built by comparative modeling based on the hP2X3R crystal structure in the closed state (PDB accession no. 5SVJ) using C3 symmetry constraints, a fragment library based on the P2X1R sequence obtained from the ROBETTA server, the target-template sequence alignment, and the boundaries of transmembrane helices. Residues 10–26 and 358–374 were added via ab initio modeling. Combining comparative and ab initio modeling used different Rosetta modules and constraint types such as RosettaMembrane (Yarov-Yarovoy et al., 2006), symmetry and distance constraints, and the RigidChunkClaimer class for constraining the extracellular and transmembrane regions during the modeling process. This was achieved using the Rosetta Broker tool (Porter et al., 2015). In additional runs, Cα-Cα distance constraints (setting: harmonic, weight 10) derived from cross-linking of cysteine mutants were included. For each run, 20,000 models were generated, scored using the Rosetta Score function, and ranked. For all runs, the 10% top-ranking structures were clustered based on the intracellular region using the hierarchical clustering option in cpptraj (Roe and Cheatham, 2013). The best-scoring structures from the largest 10 clusters were visualized and analyzed with respect to the cross-linking data. Normal mode analysis was performed for the extracellular regions and the transmembrane (TM) domain of the hP2X1R model in the closed state using iMOD with default settings (Lopéz-Blanco et al., 2011). For open/closed state comparisons, a homology model of the hP2X1R trimer in the ATP-bound, open state was built in MODELLER 9.15 (Webb and Sali, 2016) using hP2X3R (PDB accession no. 5SVK; sequence identity between hP2X1R and hP2X3R: 47%) as template.

### Online supplemental material

Table S1 contains adjusted P values from ANOVA of dimer formation. Figure S1 shows residue–residue distance distributions calculated from ab initio modeling. Video 1 illustrates dynamics of the TM helices derived from normal mode analysis. File S1 contains the P2X1R/P2X3R pairwise sequence alignment used for homology modeling.

## Results

### Cross-links are formed within one P2X receptor trimer

**Figure 1.**
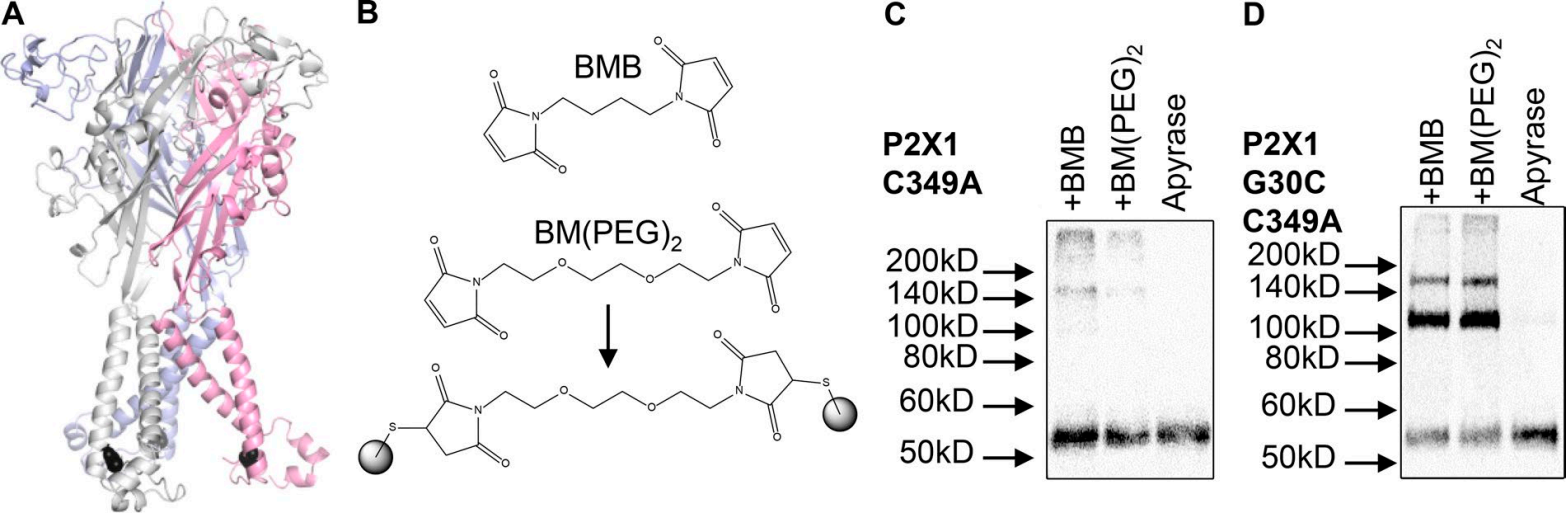
**Cysteine reactive cross-linkers dimerize subunits. (A)** Model of the hP2X1R showing the location of the G30C mutation (black spheres). The three subunits are shown in gray, light blue, and light pink. **(B)** Chemical structures of BMB and BM(PEG)_2_. The lower panel shows how BM(PEG)_2_ can cross-link two cysteine residues. **(C and D)** Representative blots from HEK293F cells transfected with hP2X1 C349A (C) and G30C C349A (D), treated with 3.2 U/ml^−1^ apyrase, then cysteine-reactive cross-linker or DMSO (apyrase only). Cells transfected with C349A (C) showed intense bands at ∼55 kD but did not show any distinct bands at ∼110 kD in any condition, indicating no dimerization of these subunits. Cells transfected with G30C C349A (D) showed robust bands at ∼55 kD and faint bands at ∼110 kD in apyrase only. The intensity of the ∼55-kD band decreased and the intensity of the ∼110-kD band increased with the addition of the cysteine cross-linkers, suggesting dimerization of the subunits.

Residue specific cross-linking compounds can be used as molecular calipers to determine distances between defined amino acids in proteins. In this study, we have used bi-functional cysteine-reactive cross-linkers with lengths (sulphhydryl to sulphhydryl) of 5.2 Å for MTS-2-MTS, 10.9 Å for BMB, and 14.7 Å for BM(PEG)_2_. In the WT hP2X1R, there are 10 cysteine residues in the extracellular loop that form five disulphide bonds and so are unavailable for cross-linking (Ennion and Evans, 2002; Kawate et al., 2009). In addition, there is a single cysteine residue in the second transmembrane region at position 349. Mutation of this cysteine to alanine (C349A) had no effect on ATP-evoked responses. When the hP2X1R is run on a Western blot, it runs predominantly as a monomer (molecular weight, ∼55 kD; Ennion and Evans, 2002). At the C349A hP2X1R mutant following treatment with the cross-linkers MTS-2-MTS, BMB, or BM(PEG)_2_ (300 µM for 30 min), there was no change in the proportion of the receptor on the gel running as a dimer (2.1 ± 0.86, 0.78 ± 0.41, 0.8 ± 0.54, and 0.6 ± 0.19% for apyrase control, MTS-2-MTS, BMB, and BM(PEG)_2_ respectively; Fig. 1). This shows that there are no free cysteine residues in the C349A mutant hP2X1R and that the C349A mutant can be used as a suitable background for testing the effects on introduced cysteine point mutants.

Under the control Western blotting conditions used, the hP2X1R was detected predominantly at ∼55 kD in accordance with the molecular weight of a glycosylated receptor monomer (Ennion et al., 2000). There was negligible dimer formation following treatment with bi-functional cysteine reactive compounds of the C349A hP2X1R consistent with a lack of free cysteine residues. However, there was ∼30% of higher-molecular-weight product. We have previously shown that the hP2X1R interacts with a range of proteins e.g., the cytoskeleton and HSP90 (Lalo et al., 2011, 2012). The high-molecular-weight product, seen following treatment of C349A hP2X1, may therefore correspond to cross-linking of interacting proteins, resulting in a more stable P2XR “macro-complex.” In addition, for some mutants (see next section), e.g., C349A K27C, there was a large amount of higher-molecular-weight product, suggesting that binding of the cross-linker to the introduced cysteine on the mutant may also directly cross-link with other cellular components.

**Figure 2.**
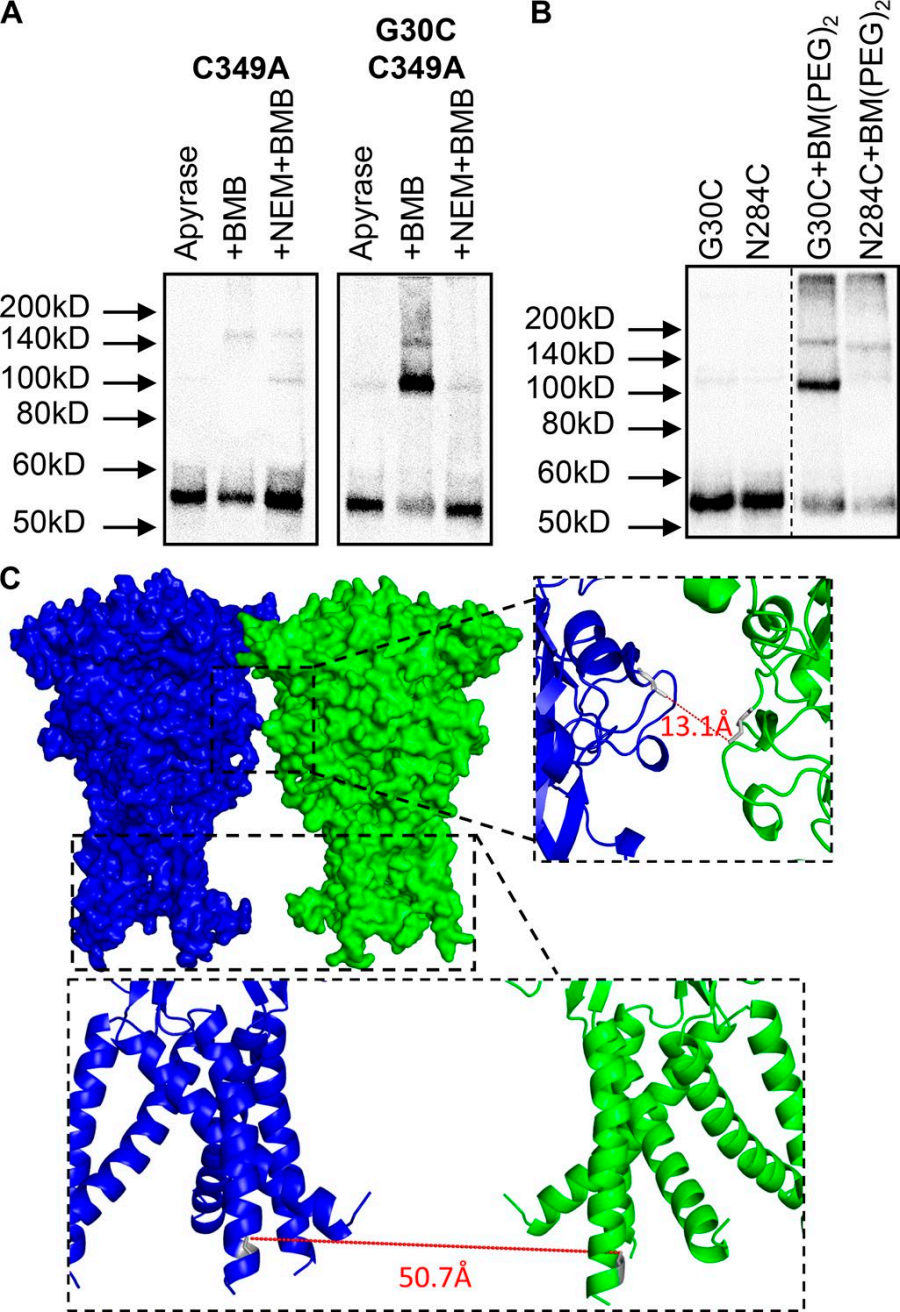
**Cross-linking can be prevented by cysteine residue blockade. (A)** Representative blots from HEK293F cells transfected with hP2X1 C349A and G30C C349A, treated with 3.2 U/ml apyrase only, apyrase then BMB, or apyrase then NEM followed by BMB. Cells transfected with C349A showed intense bands at ∼55 kD but did not show any distinct bands at ∼110 kD in any condition, indicating no dimerization of these subunits. Cells transfected with G30C C349A showed the intensity of the ∼55 kD band decreased and the intensity of the ∼110 kD band increased with the addition of BMB, suggesting dimerization of the subunits. Prior treatment with NEM showed no enhancement of the ∼110 kD band in the presence of BMB. This shows that NEM treatment prevents BMB from accessing the introduced cysteine residue and inhibits dimerization. The outline shows lanes taken from different membranes. **(B)** Example blots from cells transfected with either G30C C349A or N284C C349A, and treated with either apyrase only or apyrase with BM(PEG)_2_. The mutation N284C did not show an enhancement of the ∼110 kD band with BM(PEG)_2_ treatment, suggesting these residues cannot be cross-linked. The dotted line indicates where lanes have been removed for clarity. **(C)** Homology models of two hP2X1 N284C receptors, showing that N284C (gray sticks) from two separate receptors could be within ∼13 Å of each other without the receptors clashing, as shown by line, while G30C were ∼51 Å apart. The two models were arranged manually to minimize the N284-N284 and G30-G30 distances while keeping the central axes of both models perpendicular to the membrane plane.

From the available apo state P2XR crystal structures, there is information on the molecular distance between residues at the base of the transmembrane regions. We therefore initially tested whether the cysteine substitution at glycine 30 (G30C) could be cross-linked. G30C was chosen, as based on the hP2X3 homology model, the Cys is expected to point toward the central axis of the P2XR trimer. This in principle makes it possible to cross-link two N-terminal TM helices. The experiments were done in the presence of apyrase to break down any endogenous ATP and so keep the receptor in the apo ligand-free state. If there was cross-linking between subunits, we predict that we would now be able to detect receptor dimers on the Western blot. This was the case, demonstrating that at some point, the cysteine residues are close enough to cross-link (Fig. 1). There is also evidence of a higher-molecular-weight band at ∼160–170 kD that we suggest corresponds to the more stable association of the P2X1R trimer (under the sample preparation and gel running conditions tested) when two subunits are cross-linked. Pretreatment of the G30C C349A mutant with NEM should bind any accessible free cysteine residues and abolished the cross-linking (Fig. 2 A), confirming that the cross-linking is cysteine-dependent. Cross-linkers were applied for 30 min as this was the time recommended on the product data sheet. As a control for the effect of application time, we directly compared the cross-linking with BMB for 1 min or 30 min at the G30C C349A mutant. Following 1 min incubation, the proportion of dimer was 46 ± 7% and not different from that at 30 min (53 ± 4%, *n* = 3 for both). To determine that the dimer corresponds to fully folded and assembled, hP2X1R surface-expressed receptors were isolated using sulfo-NHS-LC-biotin. At the cell surface, the dimer accounted for 44 ± 9% (*n* = 6) of the hP2X1R and was equivalent to that for total hP2X1R protein (P = 0.23). These results suggest that the cysteine-reactive cross-linkers can be used as molecular calipers to estimate distances in hP2XRs.

We were interested to see if cross-linking had an effect on the properties of hP2X1Rs. As an initial control, we used the C349A mutant that was not cross-linked by BMB. ATP (100 µM) evoked desensitizing inward currents at the C349A hP2X1R (383 ± 140 pA, *n* = 5). However, following treatment with BMB, ATP-evoked responses were below the limit of detection. We have previously shown that hP2X1Rs are regulated by a range of intracellular proteins and intracellular factors (Lewis and Evans, 2000; Lalo et al., 2012). These results suggest that the cross-linker interferes with these interactions to inhibit function of the hP2X1R C349A receptor (that is not cross-linked), and so it was not possible to interpret whether the cross-linkers have an additional effect on the G30C C349A mutant (where ATP responses were also abolished following BMB treatment).

The cross-linking of introduced cysteine residues could lead to the formation of receptor dimers through inter- and/or intrasubunit interactions. Analysis of residue distances between two homology models of the hP2X1R suggests that it is unlikely that G30C mutants in adjacent trimers would come into close enough contact to be cross-linked as they are predicted to be ∼50 Å apart (Fig. 2). Direct fluorescent labeling of the N284C mutant with MTS-TAMRA (Fryatt and Evans, 2014) shows that asparagine residue 284 is on the surface of the receptor. Analysis of homology models shows that if adjacent receptors come into close proximity, cysteine side chains of the N284C mutants can come within ∼13 Å (Fig. 2). However, the cross-linker BM(PEG)_2_ (14.7 Å) only cross-linked the G30C mutant and not the N284C mutant (Fig. 2). Taken together, our results suggest that cross-linking at G30C under our experimental conditions is due to binding within the trimer and not between adjacent trimers.

### Cross-linking of amino-terminal residues 25–30 and carboxyl-terminal residues 355–360

**Figure 3.**
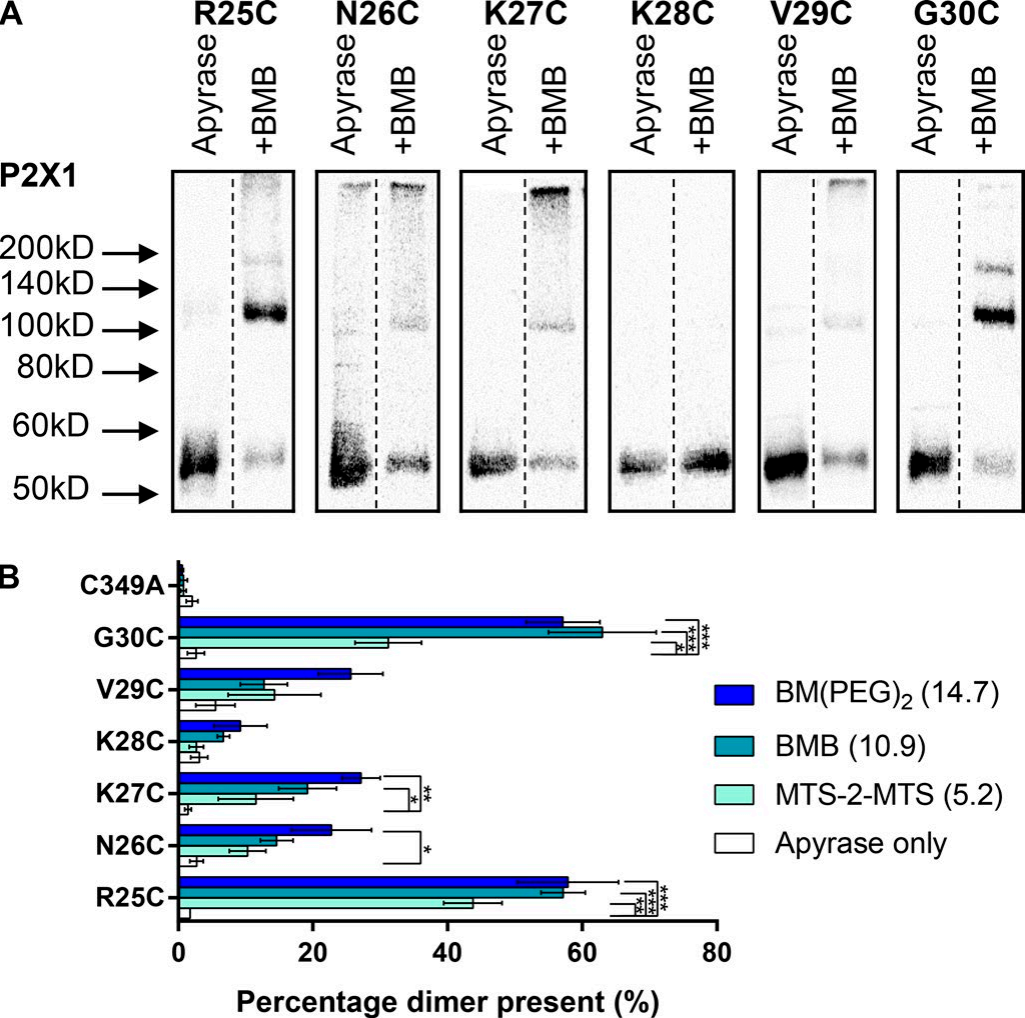
**Specific amino-terminal cysteine residues form cross-links. (A)** Representative blots from HEK293F cells transfected with hP2X1 C349A, R25C, N26C, K27C, K28C, V29C, or G30C treated with 3.2 U/ml apyrase, then BMB or DMSO (apyrase only). Of the mutants tested, only R25C and G30C showed strong enhancement of the ∼110 kD band with BMB treatments. This suggests that only R25C and G30C can dimerize within 10.9 Å. Outlines show images were taken from the same blot, while the dotted line shows where lanes have been removed for clarity. **(B)** Histogram showing the average percentage dimer present for the above mutants tested with all three cross-linkers. This shows that only R25C and G30C show ∼60% dimerization with BMB or BM(PEG)_2_ treatments. (*, P < 0.05; **, P < 0.01; ***, P < 0.001; *n* = 3–5).

Our initial studies with G30C confirmed intersubunit dimer formation. To study the intracellular juxta-membrane amino termini, we have looked in detail at residues R25-G30 with the cross-linkers MTS-2-MTS (5.2 Å), BMB (10.9 Å), and BM(PEG)_2_ (14.7 Å; Fig. 3). For all of the individual cysteine point mutants, we have previously shown that they have no effect on peak current amplitude, ATP sensitivity, and <2-fold change in the time-course of desensitization (Wen and Evans, 2009). If there is efficient cross-linking within a trimer, then 66% of the subunits (intensity on the Western blot) should be detected as a dimer. The most significant levels of cross-linking were found for G30C and R25C. At the G30C mutant, there was ∼60% dimer following treatment with BMB and BMB(PEG)_2_, indicating efficient cross-linking. With the shorter cross-linker MTS-2-MTS, ∼33% dimer was detected (31.2 ± 4.9%, P = 0.018), demonstrating that the residues at position 30 come into closer apposition less frequently. High levels of dimerization are seen for the R25C mutant for all three cross-linkers, and in this case, the shorter cross-linker MTS-2-MTS is equally effective. In contrast, there was essentially no cross-linking (≤10% of dimerization) for K28C. Dimer formation was intermediate for the remaining mutants N26C, K27C, and V29C, with a trend to greater dimer formation the longer the cross-linker.

**Figure 4.**
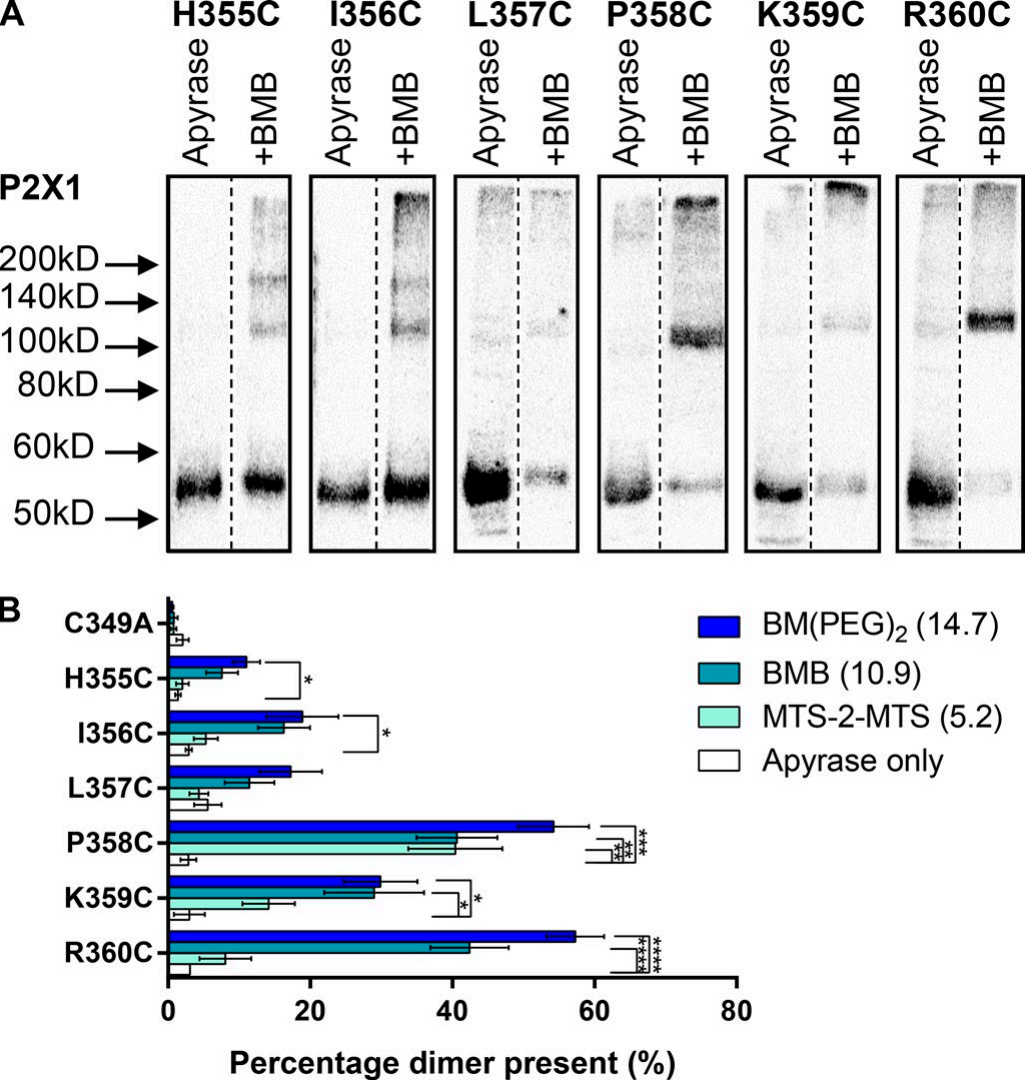
**Specific carboxyl-terminal cysteine residues form cross-links. (A)** Representative blots from HEK293F cells transfected with hP2X1 C349A H355C, I356C, L357C, P358C, K359C, or R360C treated with 3.2 U/ml apyrase, then BMB or DMSO (apyrase only). Of the mutants tested, only P358C and R360C showed strong enhancement of the ∼110 kD band with BMB treatments. This suggests that only these residues can dimerize within 10.9 Å. Boxes show images were taken from the same blot while the the dotted line shows where lanes have been removed for clarity. **(B)** Histogram showing the average percentage dimer present for the above mutants tested with all three cross-linkers. This shows that only P358C and R360C show 40–60% dimerization. (*, P < 0.05; **, P < 0.01; ***, P < 0.001; ****, P < 0.0001; *n* = 3–5).

To gain equivalent information for the intracellular juxta-membrane carboxy termini, we extended our cross-linking study to the residues H355-R360. Mutation of the individual residues in the run 355–360 had little or no effect on basic channel properties (≤2-fold decrease in peak current amplitude for H355C, K359C, and R360C (with no effect on surface expression) and no change in ATP sensitivity and little or no effect on the time-course except for R360C that showed a >2-fold increase in the rate of desensitization (unpublished data and Wen and Evans, 2011) and therefore function essentially normally. At the H355C and I356C mutants, there was a significant amount of cysteine cross-linking: ∼20% dimer with BM(PEG)_2_ (Fig. 4). No significant increase in dimerization was seen for any of the cross-linkers at L357C. At the P358C mutant, all three cross-linkers produced a significant increase in dimerization. BMB and BM(PEG)_2_, but not the shorter MTS-2-MTS, increased dimerization at K359C and R360C mutants. The most significant level of cross-linking was found for R360C.

### Cross-linking of the amino and carboxyl termini in the desensitized state

**Figure 5.**
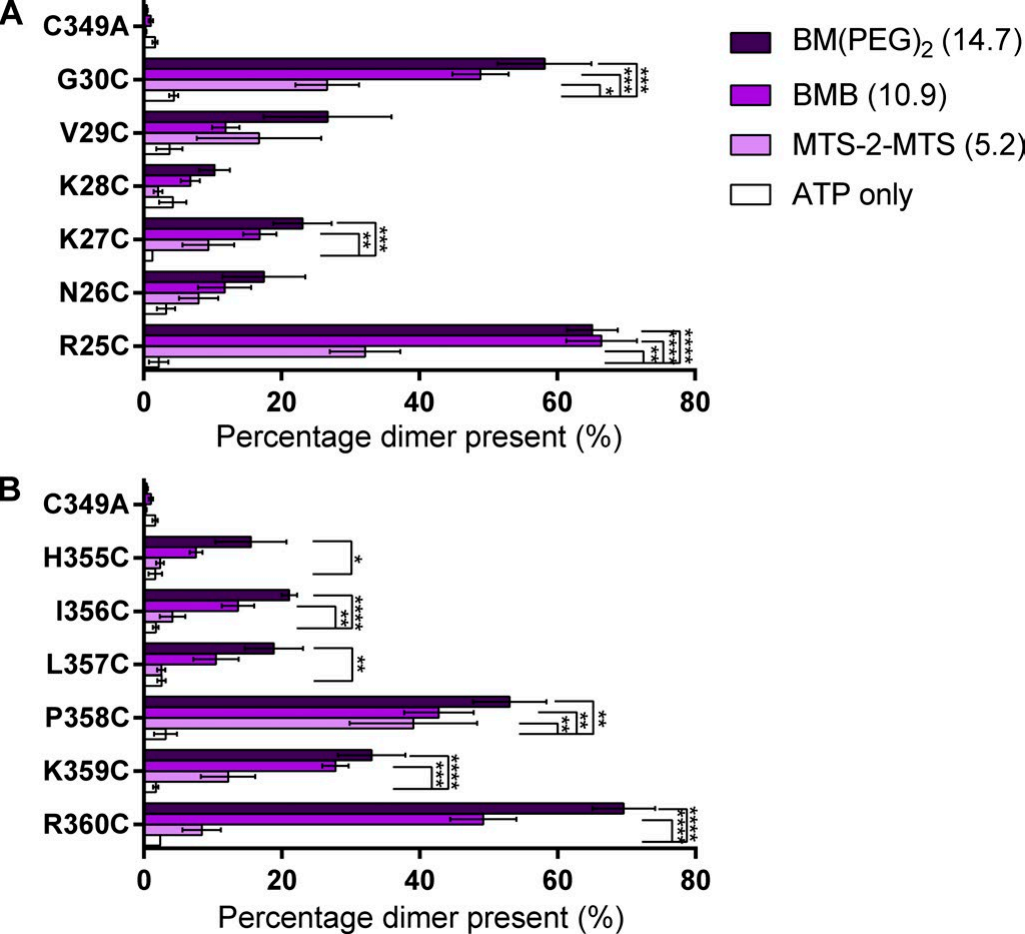
**Specific amino and carboxyl-terminal cysteine residues form cross-links with ATP pretreatment. (A)** Histogram showing the average percentage dimer present for R25C to G30C tested with all three cross-linkers in the presence of 300 µM ATP. This shows that only R25C and G30C show 40–60% dimerization. (*, P < 0.05; **, P < 0.01; ***, P < 0.001; ****, P < 0.0001, *n* = 3–5). **(B)** Histogram showing the average percentage dimer present for H355C to R360C tested with all three cross-linkers in the presence of 300 µM ATP. This shows that only P358C and R360C show 40–60% dimerization. (*, P < 0.05; **, P < 0.01; ***, P < 0.001; ****, P < 0.0001; *n* = 3–5).

The P2X1R shows desensitization within ∼1–2s of continued agonist exposure. A crystal structure of the hP2X3R is available in the desensitized state, and like the apo state, no structure associated with the intracellular regions could be resolved. To map any structural features associated with the desensitized state, we repeated the cross-linking of cysteine residues R25-G30 and H355-R360 following treatment with ATP to drive the hP2X1R into the desensitized state. The pattern of cross-linking seen was essentially the same as that for the apo form of the receptor (Fig. 5).

### Structural framework for analysis of cross-links

Structural information for extracellular and TM regions of the hP2X1R in the closed state can be derived based on homology to the closed state x-ray structure of the hP2X3R. For the intracellular termini in the closed state, no such information is available from x-ray structures, hence residues 10–26 and 358–374 were modeled ab initio in Rosetta. This results in a diverse set of clusters of models with substantial variation in the intracellular region. Common features were short elements of secondary structure; for instance, an α-helical region for residues 360–367 was found in most clusters oriented perpendicular to the membrane (albeit in different orientations) with side chains of Y362 and Y363 interacting with the membrane. For the N-terminal region, two short antiparallel β-strands (residues 13–15 and 21–23) were commonly found. The models of the intracellular regions were used to provide a range of plausible distances Cα-Cα between intracellular residues of different subunits within symmetric structures (Fig. S1) that could be compared with distances from experimental cross-links.

## Discussion

### Position specific cross-linking suggests structural organization

For the cysteine mutants R25C, G30C, and R360C treated with cross-linkers, the hP2X1R dimer accounted for ∼60% of the protein. This is consistent with the formation of an intersubunit cross-link. For some mutants, e.g., K27C and K359C, there was intermediate cross-linking, but there was no significant dimer formation for, e.g., K28C and L357C. The level of cross-linking is dependent on the location of the cysteine residue and highlights “cross-linking patterns” for the sequence stretches R25C-G30C and H355C-R360C in both the apo and desensitized states. The patterns found in R25C-G30C (Fig. 3) and H355C-R360C (Fig. 4) would not be expected if these regions were fully disordered. This suggests that there is some structural organization to both the juxta-membrane amino and carboxyl termini that favors cross-linking of some cysteine mutants and blocks cross-linking in others. The detection of organization in the intracellular domains of the hP2X1R at rest contrasts with the crystallization of the hP2X3R in the apo state (Mansoor et al., 2016). In that structure, the intracellular regions were not resolved, suggesting a flexible and disordered arrangement (Mansoor et al., 2016). However, it is important to consider that crystallization is dependent on the stability of particular conformations under the conditions used. For example, the crystallization of the β2-adrenoceptor required the inclusion of cholesterol (Cherezov et al., 2007). There is clear evidence that P2XRs are regulated by the lipid environment and cholesterol content (Bernier et al., 2013; Murrell-Lagnado, 2017). For the hP2X1R, we have previously shown that the intracellular juxta-membrane amino terminus mediated regulation of the receptor by cholesterol and the cytoskeleton (Allsopp et al., 2010; Lalo et al., 2011). Therefore, the native membrane environment of the current study may enable the stabilization of intracellular structural elements in apo and desensitized states.

### Cross-links in a structural context

Cross-linking compounds give a measure of the distances that can be bridged by the compound and can capture information on conformations that a protein can adopt. The distances may be shorter or indeed longer than derived from a particular state captured by a crystal structure. This reflects the dynamics seen in proteins that may not be fully apparent from x-ray structures that represent states most stable under the crystallization conditions. For example, Radestock and Forrest (2011) commented on the need for additional models of various states to explain experimentally derived distance constraints, which can be shorter than expected from x-ray structures. We analyzed the best supported cross-links R25C, G30C, and R360C in the context of respective distances from structural models.

**Figure 6.**
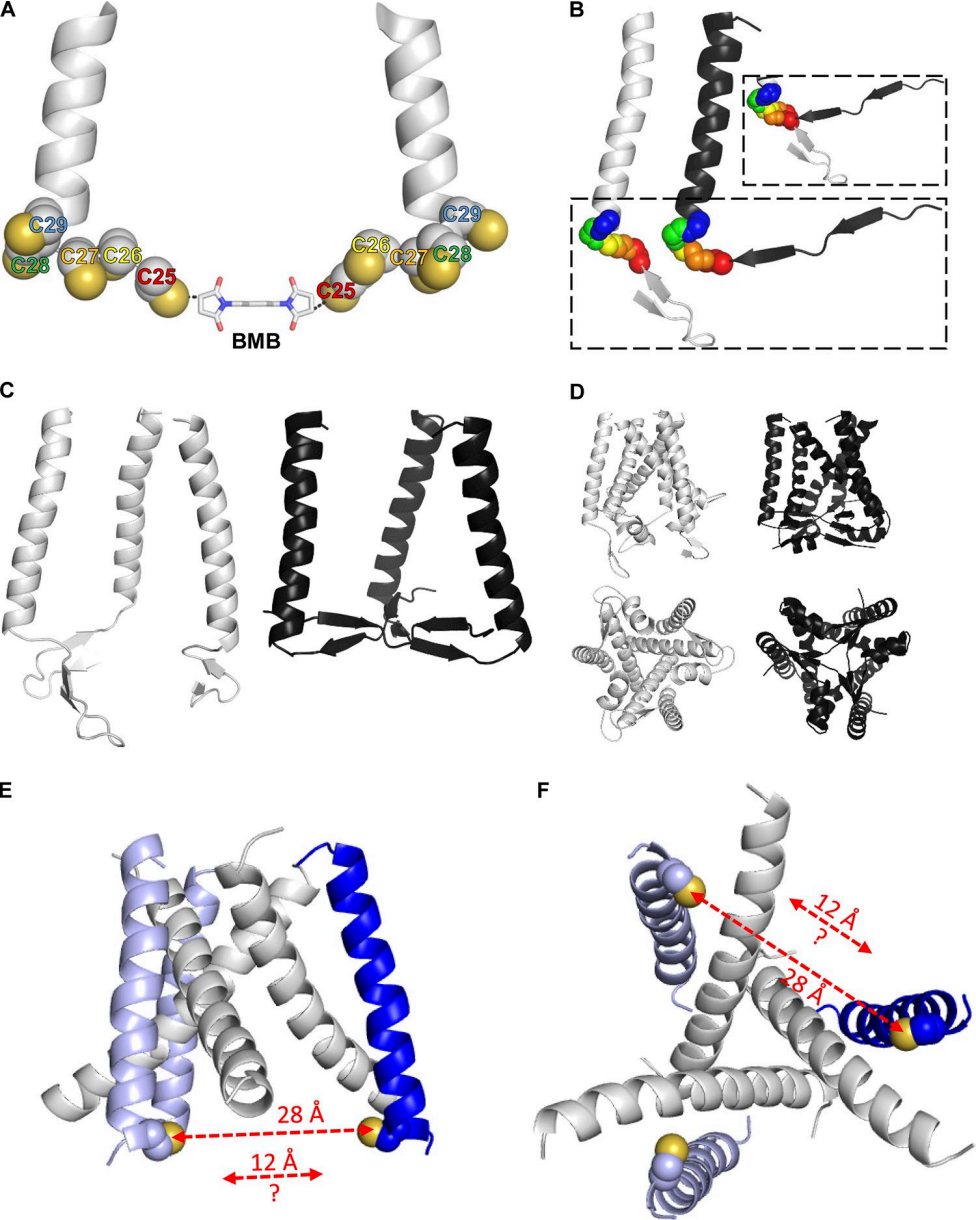
**Visualization of cross-links on structural models. (A)** TM1 of the hP2X1R in the closed state and residues 25–29 mutated to cysteines with BMB (10.9 Å) as cross-linker. **(B)** Cartoon representation of TM1 and N-terminal region (one subunit) for an ab initio model with residues 25 in cross-linking distance of the hP2X1R in the closed state (gray) and the hP2X1R in the ATP-bound state homology model (black). Main chain atoms for the residues 25–29 are shown as spheres, colored in rainbow order; zoom-in box refers to the superimposition of the N-terminal region. **(C)** Cartoon representation of TM1 and N-terminal region (trimer) for the ab initio model of the hP2X1R in the closed state (gray) and the hP2X1R in the ATP-bound state homology model (black). **(D)** Same representation as C but with TM2 and C-terminal region added. **(E)** Transmembrane region of the hP2X1R G30C homology model in the closed state viewed along the membrane. The N-terminal TM-helices are shown in blue, C-terminal TM helices in white. The C30 side-chain is shown as spheres with the cysteine sulfur atom in yellow. The red double arrows indicate the actual C30-C30 sulfur-sulfur distance as derived from the homology model (28 Å), and the C30-C30 sulfur-sulfur distance that is required to accommodate a G30C-G30C cross-link (12 Å). **(F)** As in E, but rotated by 90°, view perpendicular to the membrane from the intracellular side.

The ab initio models for the intracellular regions provide a range of Cα-Cα distances for R25C-G30C and H355C-R360C that can be compared with cross-linking distances (Fig. S1). For R25C, the shortest distances found (based on R25 Cα-Cα distance between different subunits) were 20.5 Å, equivalent to a cross-linking distance of 11.9 Å, indicating that such a cross-link is feasible within the sampled models. To enhance sampling of conformations with R25-R25 distances in the range captured by cross-links, we generated an additional set of models incorporating a 19.5 Å Cα-Cα constraint (equivalent to a 10.9-Å cross-linking distance) for R25C-R25C distances. These constraints did not introduce any additional clashes (Fig. S1), and suggest that a R25C-R25C cross-link could be accommodated within a symmetrical conformation of the P2X1R (Fig. 6). A common feature of the R25C constrained models is that the residues preceding the N-terminal TM helix are in a position more toward the central axis of the P2XR trimer. As for the unconstrained models, two antiparallel β-strands were found formed by L13-T18 and M21-R25, and an α-helix in the C-terminal region K359-K367. As one would expect for different states of the receptor, such an arrangement is different from the conformation found in the open state hP2X3R x-ray structure (Fig. 6).

G30 is part of the N-terminal TM, and the position and orientation of G30C can be derived by structural homology to hP2X3R in the closed state (PDB accession no. 5SVJ). Within the closed state hP2X1R G30C model, the distance between two sulfur atoms from G30C is ∼30 Å. To probe to what extent shorter G30C-G30C distances could be accommodated within the C3-symmetry found in the experimentally determined P2XR structures, the Rosetta modeling process was rerun with distance constraints on all G30 pairs within hP2X1R. This approach did not result in any physically meaningful models as indicated by increased Rosetta scores compared with the unconstrained model (Fig. S1). Structurally, any substantial concerted inward movement of the N-terminal TM helices is limited by the positioning of the C-terminal TM helices, and these have little space for tighter packing in the center of the pore. To explore whether the G30C-G30C cross-link might capture a transient dynamic movement of N-terminal TM helices, we performed normal mode analysis of the hP2X1R homology model based on the closed state of hP2X3R. Indeed, the first normal mode shows movement of the TM helices relative to each other, thereby reducing one of the G30C-G30C distances by ∼5 Å (Video 1). However, the extent of this movement is not sufficient to explain the actual cross-link (Fig. 6), indicating that the TM domain may be more dynamic/less stable than one might expect from the static x-ray structures. Unfolding of the entire N-terminal region could explain the G30C-G30C cross-link. However, this seems unlikely, as then one would expect to see, e.g., V29C-V29C cross-linking, and this is not found. How far the observed G30C-G30C cross-link formation reflects transient localized unfolding around G30C, protein dynamics, “capturing a rare event,” or a combination thereof remains to be determined.

The minimum distance between two R360C residues (Cα-Cα) in the symmetrical unconstrained models of the intracellular region is 40.5 Å (equivalent to a cross-link distance of 31.9 Å). Enforcing Cα-Cα distances of 23.3 Å (equivalent to cross-link distance 14.7 Å) increased the Rosetta Score substantially, indicating that such structures are physically unlikely (Fig. S1). This points to a scenario where the R360C cross-link may reflect a transient conformation not sampled in the constrained ab initio modeling.

### Functional implications

One of the key findings from the crystallization of the hP2X3R was the detection of the intracellular cap in an ATP-bound form of the receptor with the channel gate open. The cap forms from complex interactions of the intracellular termini and has been described to be important for/as a conduit for ionic permeation. This is consistent with several previous mutagenesis studies. For example, for the hP2X1R, residues 27–29 when changed to the equivalent hP2X2R residues produced a slowing in desensitization (Allsopp and Evans, 2011). At the C terminus, swapping the region from R360 to Q365 for those in the hP2X2R made desensitization of the hP2X1R even faster, and recovery of the hP2X1R R360N mutant was 10-fold longer than for the WT (Allsopp et al., 2013). In addition, voltage clamp fluorometry studies that measured real-time movement in the extracellular ligand binding domain of the hP2X1R demonstrated that rearrangements in the extracellular domain on agonist washout occurred more slowly than the recovery from desensitization (Fryatt and Evans, 2014). These findings suggest that the differences seen, e.g., in desensitization kinetics above are a consequence of the dynamics of cap formation, and the equilibrium of states and conformations in the intracellular region.

For efficient channel gating, the intracellular cap of the open state needs to fold quickly and efficiently. One way for this to be facilitated would be by having preformed elements in the apo state of the receptor. Our data indeed indicate that such a “precap” structure may exist for the hP2X1R. Similar considerations apply to the ATP-bound desensitized state. In the respective hP2X3R x-ray structure (PDB accession no. 5SVL), the channel gate adopts a closed structure, and the cytoplasmic cap or, indeed, any structural organization of the intracellular domains could not be detected. In the current study, we were able to detect essentially the same cross-linking pattern in the desensitized receptor as found for the apo form of the receptor, which indicates there may be a “postcap” organization of the intracellular juxta-membrane regions in the desensitized state. A functional model for the intracellular region emerges where ATP-induced conformational change and rigidification of the extracellular domain are followed by opening of the channel and “full” cytoplasmic cap formation. On desensitization, the channel closes and the cytoplasmic cap transitions to the “postcap” state. Following ATP washout, the receptor then returns to the “precap” apo state.

In summary, our data suggest that when expressed in the native membrane environment of HEK cells, there is structural organization of the intracellular domains of the hP2X1R in the apo and desensitized states that is likely to contribute to the efficient gating of the channel. Future work will need to establish the intricate nature of states and transitions in the intracellular region. Recent development of techniques using artificial fluorescent amino acids may enable the use of voltage clamp fluorometry to study simultaneously the interplay between local structural rearrangements and functional electrophysiology in the native membrane environment in real time (Gordon et al., 2018).

## Supplementary Material

Supplemental Materials (PDF)

Video 1

Sequence alignment (.txt)

## References

[bib1] Abdulqawi R., Dockry R., Holt K., Layton G., McCarthy B.G., Ford A.P., and Smith J.A. 2015 P2X3 receptor antagonist (AF-219) in refractory chronic cough: a randomised, double-blind, placebo-controlled phase 2 study. Lancet. 385:1198–1205. 10.1016/S0140-6736(14)61255-1 25467586

[bib2] Allsopp R.C., and Evans R.J. 2011 The intracellular amino terminus plays a dominant role in desensitization of ATP-gated P2X receptor ion channels. J. Biol. Chem. 286:44691–44701. 10.1074/jbc.M111.303917 22027824 PMC3247974

[bib3] Allsopp R.C., and Evans R.J. 2015 Contribution of the Juxtatransmembrane Intracellular Regions to the Time Course and Permeation of ATP-gated P2X7 Receptor Ion Channels. J. Biol. Chem. 290:14556–14566. 10.1074/jbc.M115.642033 25903136 PMC4505523

[bib4] Allsopp R.C., Lalo U., and Evans R.J. 2010 Lipid raft association and cholesterol sensitivity of P2X1-4 receptors for ATP: chimeras and point mutants identify intracellular amino-terminal residues involved in lipid regulation of P2X1 receptors. J. Biol. Chem. 285:32770–32777. 10.1074/jbc.M110.148940 20699225 PMC2963349

[bib5] Allsopp R.C., Farmer L.K., Fryatt A.G., and Evans R.J. 2013 P2X receptor chimeras highlight roles of the amino terminus to partial agonist efficacy, the carboxyl terminus to recovery from desensitization, and independent regulation of channel transitions. J. Biol. Chem. 288:21412–21421. 10.1074/jbc.M113.464651 23740251 PMC3774408

[bib6] Bernier L.P., Ase A.R., and Séguéla P. 2013 Post-translational regulation of P2X receptor channels: modulation by phospholipids. Front. Cell. Neurosci. 7:226 10.3389/fncel.2013.00226 24324400 PMC3838964

[bib7] Boué-Grabot E., Archambault V., and Séguéla P. 2000 A protein kinase C site highly conserved in P2X subunits controls the desensitization kinetics of P2X(2) ATP-gated channels. J. Biol. Chem. 275:10190–10195. 10.1074/jbc.275.14.10190 10744703

[bib8] Brändle U., Spielmanns P., Osteroth R., Sim J., Surprenant A., Buell G., Ruppersberg J.P., Plinkert P.K., Zenner H.-P., and Glowatzki E. 1997 Desensitization of the P2X(2) receptor controlled by alternative splicing. FEBS Lett. 404:294–298. 10.1016/S0014-5793(97)00128-2 9119082

[bib9] Chaumont S., Compan V., Toulme E., Richler E., Housley G.D., Rassendren F., and Khakh B.S. 2008 Regulation of P2X2 receptors by the neuronal calcium sensor VILIP1. Sci. Signal. 1:ra8 10.1126/scisignal.1162329 18922787 PMC3523710

[bib10] Cherezov V., Rosenbaum D.M., Hanson M.A., Rasmussen S.G., Thian F.S., Kobilka T.S., Choi H.J., Kuhn P., Weis W.I., Kobilka B.K., and Stevens R.C. 2007 High-resolution crystal structure of an engineered human beta2-adrenergic G protein-coupled receptor. Science. 318:1258–1265. 10.1126/science.1150577 17962520 PMC2583103

[bib11] Das R., and Baker D. 2008 Macromolecular modeling with rosetta. Annu. Rev. Biochem. 77:363–382. 10.1146/annurev.biochem.77.062906.171838 18410248

[bib12] Ennion S.J., and Evans R.J. 2001 Agonist-stimulated internalisation of the ligand-gated ion channel P2X(1) in rat vas deferens. FEBS Lett. 489:154–158. 10.1016/S0014-5793(01)02102-0 11165241

[bib13] Ennion S.J., and Evans R.J. 2002 Conserved cysteine residues in the extracellular loop of the human P2X(1) receptor form disulfide bonds and are involved in receptor trafficking to the cell surface. Mol. Pharmacol. 61:303–311. 10.1124/mol.61.2.303 11809854

[bib14] Ennion S., Hagan S., and Evans R.J. 2000 The role of positively charged amino acids in ATP recognition by human P2X1 receptors. J. Biol. Chem. 275:29361 10.1074/jbc.M003637200 11063753

[bib15] Fryatt A.G., and Evans R.J. 2014 Kinetics of conformational changes revealed by voltage-clamp fluorometry give insight to desensitization at ATP-gated human P2X1 receptors. Mol. Pharmacol. 86:707–715. 10.1124/mol.114.095307 25296688

[bib16] Fujiwara Y., and Kubo Y. 2006 Regulation of the desensitization and ion selectivity of ATP-gated P2X2 channels by phosphoinositides. J. Physiol. 576:135–149. 10.1113/jphysiol.2006.115246 16857707 PMC1995631

[bib17] Gordon S.E., Munari M., and Zagotta W.N. 2018 Visualizing conformational dynamics of proteins in solution and at the cell membrane. eLife. 7:e37248 10.7554/eLife.37248 29923827 PMC6056233

[bib18] Hattori M., and Gouaux E. 2012 Molecular mechanism of ATP binding and ion channel activation in P2X receptors. Nature. 485:207–212. 10.1038/nature11010 22535247 PMC3391165

[bib19] Hausmann R., Bahrenberg G., Kuhlmann D., Schumacher M., Braam U., Bieler D., Schlusche I., and Schmalzing G. 2014 A hydrophobic residue in position 15 of the rP2X3 receptor slows desensitization and reveals properties beneficial for pharmacological analysis and high-throughput screening. Neuropharmacology. 79:603–615. 10.1016/j.neuropharm.2014.01.010 24452010

[bib20] Hechler B., Lenain N., Marchese P., Vial C., Heim V., Freund M., Cazenave J.-P., Cattaneo M., Ruggeri Z.M., Evans R., and Gachet C. 2003 A role of the fast ATP-gated P2X1 cation channel in thrombosis of small arteries in vivo. J. Exp. Med. 198:661–667. 10.1084/jem.20030144 12913094 PMC2194166

[bib21] Kaczmarek-Hájek K., Lörinczi E., Hausmann R., and Nicke A. 2012 Molecular and functional properties of P2X receptors--recent progress and persisting challenges. Purinergic Signal. 8:375–417. 10.1007/s11302-012-9314-7 22547202 PMC3360091

[bib22] Kawate T., Michel J.C., Birdsong W.T., and Gouaux E. 2009 Crystal structure of the ATP-gated P2X(4) ion channel in the closed state. Nature. 460:592–598. 10.1038/nature08198 19641588 PMC2720809

[bib23] Kim M., Jiang L.-H., Wilson H.L., North R.A., and Surprenant A. 2001 Proteomic and functional evidence for a P2X7 receptor signalling complex. EMBO J. 20:6347–6358. 10.1093/emboj/20.22.6347 11707406 PMC125721

[bib24] Lalo U., Roberts J.A., and Evans R.J. 2011 Identification of human P2X1 receptor-interacting proteins reveals a role of the cytoskeleton in receptor regulation. J. Biol. Chem. 286:30591–30599. 10.1074/jbc.M111.253153 21757694 PMC3162419

[bib25] Lalo U., Jones S., Roberts J.A., Mahaut-Smith M.P., and Evans R.J. 2012 Heat shock protein 90 inhibitors reduce trafficking of ATP-gated P2X1 receptors and human platelet responsiveness. J. Biol. Chem. 287:32747–32754. 10.1074/jbc.M112.376566 22851178 PMC3463321

[bib26] Lecut C., Faccinetto C., Delierneux C., van Oerle R., Spronk H.M., Evans R.J., El Benna J., Bours V., and Oury C. 2012 ATP-gated P2X1 ion channels protect against endotoxemia by dampening neutrophil activation. J. Thromb. Haemost. 10:453–465. 10.1111/j.1538-7836.2011.04606.x 22212928

[bib27] Lewis C.J., and Evans R.J. 2000 Lack of run-down of smooth muscle P2X receptor currents recorded with the amphotericin permeabilized patch technique, physiological and pharmacological characterization of the properties of mesenteric artery P2X receptor ion channels. Br. J. Pharmacol. 131:1659–1666. 10.1038/sj.bjp.0703744 11139444 PMC1572503

[bib28] Lopéz-Blanco J.R., Garzón J.I., and Chacón P. 2011 iMod: multipurpose normal mode analysis in internal coordinates. Bioinformatics. 27:2843–2850. 10.1093/bioinformatics/btr497 21873636

[bib29] Mansoor S.E., Lü W., Oosterheert W., Shekhar M., Tajkhorshid E., and Gouaux E. 2016 X-ray structures define human P2X(3) receptor gating cycle and antagonist action. Nature. 538:66–71. 10.1038/nature19367 27626375 PMC5161641

[bib30] Murrell-Lagnado R.D. 2017 Regulation of P2X Purinergic Receptor Signaling by Cholesterol. Curr. Top. Membr. 80:211–232. 10.1016/bs.ctm.2017.05.004 28863817

[bib31] Porter J.R., Weitzner B.D., and Lange O.F. 2015 A Framework to Simplify Combined Sampling Strategies in Rosetta. PLoS One. 10:e0138220 10.1371/journal.pone.0138220 26381271 PMC4575156

[bib32] Radestock S., and Forrest L.R. 2011 The alternating-access mechanism of MFS transporters arises from inverted-topology repeats. J. Mol. Biol. 407:698–715. 10.1016/j.jmb.2011.02.008 21315728

[bib33] Roberts J.A., and Evans R.J. 2007 Cysteine substitution mutants give structural insight and identify ATP binding and activation sites at P2X receptors. J. Neurosci. 27:4072–4082. 10.1523/JNEUROSCI.2310-06.2007 17428985 PMC2092412

[bib34] Roe D.R., and Cheatham T.E. III 2013 PTRAJ and CPPTRAJ: Software for Processing and Analysis of Molecular Dynamics Trajectory Data. J. Chem. Theory Comput. 9:3084–3095. 10.1021/ct400341p 26583988

[bib35] Roger S., Gillet L., Baroja-Mazo A., Surprenant A., and Pelegrin P. 2010 C-terminal calmodulin-binding motif differentially controls human and rat P2X7 receptor current facilitation. J. Biol. Chem. 285:17514–17524. 10.1074/jbc.M109.053082 20378545 PMC2878516

[bib36] Simon J., Kidd E.J., Smith F.M., Chessell I.P., Murrell-Lagnado R., Humphrey P.P.A., and Barnard E.A. 1997 Localization and functional expression of splice variants of the P2X2 receptor. Mol. Pharmacol. 52:237–248. 10.1124/mol.52.2.237 9271346

[bib37] Surprenant A., and North R.A. 2009 Signaling at purinergic P2X receptors. Annu. Rev. Physiol. 71:333–359. 10.1146/annurev.physiol.70.113006.100630 18851707

[bib38] Watson P.J., Millard C.J., Riley A.M., Robertson N.S., Wright L.C., Godage H.Y., Cowley S.M., Jamieson A.G., Potter B.V., and Schwabe J.W. 2016 Insights into the activation mechanism of class I HDAC complexes by inositol phosphates. Nat. Commun. 7:11262 10.1038/ncomms11262 27109927 PMC4848466

[bib39] Webb B., and Sali A. 2016 Comparative Protein Structure Modeling Using MODELLER. Curr. Protoc. Protein Sci. 86:2.9.1–2.9: 37. 10.1002/cpps.20 27801516

[bib40] Wen H., and Evans R.J. 2009 Regions of the amino terminus of the P2X receptor required for modification by phorbol ester and mGluR1alpha receptors. J. Neurochem. 108:331–340. 10.1111/j.1471-4159.2008.05761.x 19046321 PMC2704932

[bib41] Wen H., and Evans R.J. 2011 Contribution of the intracellular C terminal domain to regulation of human P2X1 receptors for ATP by phorbol ester and Gq coupled mGlu(1α) receptors. Eur. J. Pharmacol. 654:155–159. 10.1016/j.ejphar.2010.11.039 21172341 PMC3036795

[bib42] Werner P., Seward E.P., Buell G.N., and North R.A. 1996 Domains of P2X receptors involved in desensitization. Proc. Natl. Acad. Sci. USA. 93:15485–15490. 10.1073/pnas.93.26.15485 8986838 PMC26431

[bib43] Yarov-Yarovoy V., Schonbrun J., and Baker D. 2006 Multipass membrane protein structure prediction using Rosetta. Proteins. 62:1010–1025. 10.1002/prot.20817 16372357 PMC1479309

[bib44] Young M.T., Pelegrin P., and Surprenant A. 2007 Amino acid residues in the P2X7 receptor that mediate differential sensitivity to ATP and BzATP. Mol. Pharmacol. 71:92–100. 10.1124/mol.106.030163 17032903

