# Supplementary material for: Organization of ATP-gated P2X1 receptor intracellular termini in apo and desensitized states
Source: J Gen Physiol. 2019 Feb 4;151(2):146–55. doi: 10.1085/jgp.201812108 (PMC6363416; doi:10.1085/jgp.201812108)
Supplement: Supplemental Materials (PDF) [file JGP_201812108_sm.pdf]

Supplemental material

Fryatt et al., https://10.1085/jgp.201812108

The sequence alignment used in homology modeling of the hP2X1R is provided as a .txt file.

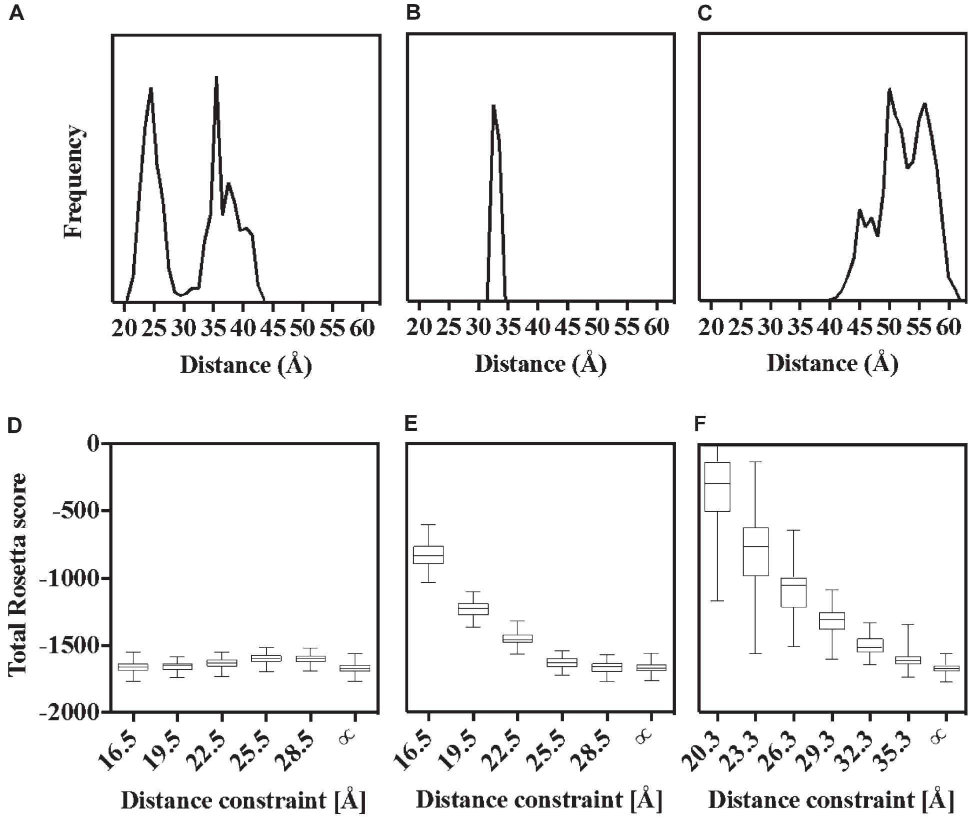

Figure S1. **Distance distribution in the unconstrained ab initio modeling run for the residues R25, G30, and R360, and total Rosetta scores with R25, G30, and R360 constraint symmetrical modeling. (A–C)** Distance distribution of the residues R25, G30, and R360 among three chains (Cα-Cα atoms), respectively. These distances were measured in the ab initio modeling run of the hP2X1R in the closed state without any cross-linking constraints. **(D–F)** Total Rosetta scores comparison for the R25, G30, and R360 constraint runs. Infinity symbol refers to the unconstrained modeling.

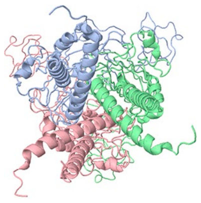

Video 1. **Normal mode analysis of P2X1R model in the closed state.** The animation shows mode 1, illustrating the possibility of asymmetric movement of the N-terminal TM.

Table S1. **Adjusted P values from the ANOVA analysis of percentage dimer formation with the cysteine reactive cross-linkers**

**Mutant**	**Adjusted P value Apyrase versus**			**No.**
	**MTS-2-MTS**	**BMB**	**BM(PEG)$_2$**	
R25C C349A	0.0013	0.0002	0.0002	3
N26C C349A	0.93	0.21	0.01	4
K27C C349A	0.48	0.027	0.0013	5
K28C C349A	>0.99	>0.99	0.51	3
V29C C349A	>0.99	>0.99	0.069	4
G30C C349A	0.0184	<0.0001	<0.0001	3
C349A H355C	>0.99	0.13	0.014	3
C349A I356C	>0.99	0.12	0.049	3
C349A L357C	>0.99	>0.99	0.094	5
C349A P358C	0.0047	0.0045	0.0006	3
C349A K359C	0.79	0.016	0.013	4
C349A R360C	>0.99	<0.0001	<0.0001	4
**Mutant**	**Adjusted P value ATP versus**			**No.**
	**MTS-2-MTS**	**BMB**	**BM(PEG)$_2$**	
R25C C349A	0.0027	<0.0001	<0.0001	3
N26C C349A	>0.99	0.45	0.073	4
K27C C349A	0.24	0.0078	0.0004	5
K28C C349A	>0.99	0.93	0.10	3
V29C C349A	0.57	>0.99	0.091	4
G30C C349A	0.027	0.0004	0.0001	3
C349A H355C	>0.99	0.47	0.019	3
C349A I356C	0.96	0.0024	<0.0001	3
C349A L357C	>0.99	0.17	0.0018	5
C349A P358C	0.0082	0.0046	0.0011	3
C349A K359C	0.13	0.0003	<0.0001	4
C349A R360C	0.79	<0.0001	<0.0001	4

Values shown were generated from multiple comparisons and reported P values from comparing ATP-treated cells against cross-linker–treated cells.
